# Distributed Leadership and New Generation Employees’ Proactive Behavior: Roles of Idiosyncratic Deals and Meaningfulness of Work

**DOI:** 10.3389/fpsyg.2021.755513

**Published:** 2021-11-10

**Authors:** Shuhui Xu, Haomin Zhang, Yi Dai, Jun Ma, Ledi Lyu

**Affiliations:** ^1^School of Management, Shanghai University, Shanghai, China; ^2^Business School, Shanghai Dianji University, Shanghai, China

**Keywords:** distributed leadership, idiosyncratic deals, meaningfulness of work, proactive behavior, multistep mediation

## Abstract

New generation employees have become the main force of the organization, and their proactive behavior directly affects the organization’s future development. How to effectively stimulate the proactive behavior of new generation employees has become a hot topic in the field of organizational management. Based on the integrated perspective of social exchange and self-enhancement, we constructed a multistep mediation model to explore the influence mechanism of distributed leadership on the proactive behavior of new generation employees. We designed a three-stage research method of supervisor-employee pairing to collect data from 26 supervisors and 304 new generation employees in a new energy vehicle company in East China. Results indicated that (a) distributed leadership is positively related to proactive behavior of new generation employees; (b) idiosyncratic deals and meaningfulness of work mediated the linkage between distributed leadership and new generation employees’ proactive behavior; (c) idiosyncratic deals and meaningfulness of work play a multistep mediation role between distributed leadership and new generation employees’ proactive behavior. These findings have theoretical implications for the proactive behavior literature and managerial implications for practitioners.

## Introduction

New generation employees born in the “post 1980s” and “post 1990s” have gradually become the backbone and protagonist of a company, and their proactive behavior directly affects the company’s future development (Fang et al., 2019). In this vein, organizations enhance the requirement of employees’ proactive behavior. Managers expect employees to break the work limits, and independently identify, analyze, and solve problems, to help organizations resist external risks and maintain a competitive advantage (Riivari et al., 2020). However, with the economic development of society and changes in work methods, the traditional authoritative style of leadership can no longer adapt to the psychological characteristics of new generation employees, who use new work concepts, techniques, and social rules (Fang et al., 2019). Therefore, how to effectively managing new generation employees and fully inspiring their proactive behavior has become a new challenge for the organization managers.

Parker et al. (2010) argue that proactive behavior refers to changes made by individuals, which needs organizational and individual power and support. The motivation for new generation employees’ proactive behavior largely depends on the leadership style of the leader (Fang et al., 2019). Distributed leadership is based on the concept of “respect and empowerment” in organizational management and it can adapt to the independent needs of new generation employees. Distributed leadership differs from other types of leadership where it emphasizes leadership as a practice that focuses on influence and agency through interpersonal interactions rather than formal roles, responsibilities, and actions, which has significant advantages in optimizing organizational decision-making (Quek et al., 2021). Distributed leadership means that leaders and employees can exchange roles according to the characteristics of tasks at different stages, and individuals with heterogeneous knowledge and skills could dynamically assume and replace leadership functions (Bolden, 2011).

There are many studies regarding the results of distributed leadership. In education research, scholars have found that distributed leadership positively affects teachers’ attitudes and behaviors (e.g., teachers’ job satisfaction, self-efficacy, and organizational commitment; Sun and Xia, 2018; Liu and Werblow, 2019). In organizational management research, scholars have proven that distributed leadership has positive impacts on individuals, teams, and organizations (e.g., it promotes employees’ taking charge behavior, team performance, and organizational change; Wang et al., 2014; Butler and Tregaskis, 2018; Canterino et al., 2020). Despite these encouraging findings (Crant, 2000; Den Hartog and Belschak, 2012; Lan et al., 2020; Bohlmann and Zacher, 2021), a notable omission is the proactive behavior of new generation employees. Therefore, the first goal of this research is to explore how distributed leadership can effectively promote the proactive behavior of new generation employees.

Parker et al. (2010) pointed out that an individual mainly considers two motivational states (“can do” and “reason to”) when deciding whether to implement proactive behavior. Based on the proactive behavior incentive model, the second goal of this study was to analyze the influence mechanism of distributed leadership on new generation employees’ proactive behavior. First, in the workplace, new generation employees generally have needs for self-growth and development, which means they need a condition for interacting with leaders and organization contexts. In this vein, idiosyncratic deals, as work agreements reached by employees and leaders through negotiation (Huang and Chen, 2021), play an important role motivating employees’ initiative (Hornung et al., 2010). Idiosyncratic deals embody employee-oriented leadership behaviors (Hornung et al., 2011), and are affected by social culture, organizational flexibility, and active family support for employees’ work (Tang and Hornung, 2015; Liao et al., 2016; Tuan, 2016). In addition, idiosyncratic deals also affect employees’ attitudes and behaviors (Wang et al., 2018; Ding and Chang, 2020; Kong et al., 2020). Liao et al. (2016) proposed that leadership style affects the negotiation of idiosyncratic deals. In the context of distributed leadership, each member plays an important and unique role (Torres, 2019). Distributed leadership is not only a tool for collecting scattered and professional knowledge, but also a means for further cultivating talent and tapping the potential of organizations. Obviously, previous studies have neglected the mediating role of idiosyncratic deals between distributed leadership and proactive behavior. According to social exchange theory, individuals will repay the value obtained from social interaction by positive behaviors that are beneficial to the counterparty. Therefore, in terms of the “can do” path, we propose the path of “distributed leadership influences proactive behavior through idiosyncratic deals.”

Second, according to self-enhancement theory, individuals generally have the demand for self-enhancement (Alicke and Sedikides, 2009; Hepper et al., 2010), that is, the psychological motivation to construct and maintain positive self-intentions (Kwang and Swann, 2010; Vecchione et al., 2013). Generally, meaningful work can be understood as a fundamental human need, which all persons require to satisfy their inescapable interests in freedom, autonomy, and dignity (Yeoman, 2014). In reality, new generation employees have special pursuits for meaningfulness of work tasks and positive self-recognition (Mihelič and Aleksić, 2017). In this vein, distributed leadership stimulates intrinsic motivation by enhancing employees’ positive awareness and affirmation of meaningfulness of work (Carton, 2018), inducing them to engage in proactive behavior. Scholars have explored the measurement methods of the meaningfulness of work (Lysova et al., 2019), dimensions (Steger et al., 2012), influencing factors (such as decent work and hope; Allan et al., 2019a,b), and consequences (such as happiness, exhaustion, and engagement Martela and Pessi, 2018; Bailey et al., 2019; Vogel et al., 2020). Some scholars are concerned that the meaningfulness of work as a boundary condition affects the impact of context on employee’ positive behavior (Sa’nchez-Cardona et al., 2020; Sawhney et al., 2020). However, empirical research on the role of meaningfulness of work as mediating role between distributed leadership and new generation employees’ proactive behavior is very rare. Therefore, in terms of the “reason to” motivational state, we propose the path of “distributed leadership influences proactive behavior through meaningfulness of work.” Furthermore, idiosyncratic deals are not only channels for exchanging important resources with employers, but may also create greater sense of value and meaningfulness (Ding and Chang, 2020). Because social exchange is an established approach to studying idiosyncratic deals, we predict incremental effects of the self-enhancement approach: “distributed leadership influences proactive behavior through idiosyncratic deals and meaningfulness of work.” The third goal of this research is to test a multistep mediation model.

We used empirical research methods to carry out research work, and found that distributed leadership positively influenced new generation employees’ proactive behavior through idiosyncratic deals and meaningfulness of work. In summary, this research has made contributions in three aspects. First, we have contributed to the literature about distributed leadership by studying the proactive behavior of new generation employees. We promoted the theoretical research of distributed leadership in organizational management research. Second, we conducted theoretical analysis and testing on the mediating roles of idiosyncratic deals and meaningfulness of work. We provided insights into how managers can stimulate the proactive behavior of new generation employees through the two paths of “can do” (sign agreements) and “reason to” (create and enhance meaningfulness). Third, we explored the multistep mediation role of idiosyncratic deals and meaningfulness of work between distributed leadership and new generation employees’ proactive behavior through the integrated perspective of social exchange and self-enhancement. Our research enriches and expands the content of previous research on the proactive behavior incentive model, and provides guidance for managers to promote the proactive behavior of new generation employees from the two paths of “can do” and “reason to.”

## Conceptual Framework and Research Hypotheses

### Distributed Leadership and New Generation Employees’ Proactive Behavior

According to social exchange theory, distributed leadership allows new generation employees to assume leadership functions dynamically based on their heterogeneous knowledge and skills, which generates a basis for reciprocity for the organization and employees. This positive leadership style, in turn, promotes new generation employees’ proactive behavior. First, the “principle of reciprocity” in social exchange theory emphasizes that employees who are recognized and supported by the organization will have a sense of reward, making positive behaviors to achieve organizational goals (Gouldner, 1960). Previous studies have shown that new generation employees prefer to actively undertake tasks rather than blindly accept a superior’s orders (Fang et al., 2019). This makes the role exchange of leaders and employees affect new generation employees’ job satisfaction and positive work attitudes (Liu et al., 2013; Sun and Xia, 2018). Positive work attitudes are conducive to promoting positive behaviors, and new generation employees will make an effort to burden proactive behavior, which can improve the organizational environment to repay the psychological benefits gained from the organization.

Furthermore, because new generation employees who advocate freedom and equality prefer a relaxed, free, and active working atmosphere, distributed leadership emphasizing shared goals and responsibilities will create an organizational atmosphere of trust, respect, and cooperation for members, enhancing new generation employee’ perceived organizational support (Yang and Yang, 2020). It is worth noting that perceived organizational support is an important condition for stimulating employees’ proactive behavior (Parker et al., 2006). According to social exchange theory, new generation employees with a high sense of organizational support will actively perform the “reciprocal obligation” and actively undertake proactive behavior to improve the organizational environment (Liu et al., 2013). Finally, new generation employees pursue self-direction and self-management, emphasizing their value in career development. At the same time, a distributed leader will focus on the value of each member, and encourage members to use their knowledge and skills to identify and deal with organizational problems. An open and self-organized leadership style increases the extra work engagement of new generation employees, providing the necessary prerequisites for their proactive behavior (Chiaburu et al., 2014). Therefore, we propose the following hypothesis:

*Hypothesis* 1: Distributed leadership has a positive impact on new generation employees’ proactive behavior.

### Distributed Leadership and Idiosyncratic Deals

Social interaction will bring individuals benefit and value, enhancing their sense of identity, degree of attachment, and willingness to maintain an autonomous relationship with the counterparty of social interaction (Gouldner, 1960). When an organization is willing to invest in the growth of employees, employees will make corresponding efforts in return for the organization. Idiosyncratic deals are non-standardized work agreements reached by employees and organizations based on voluntary negotiation (e.g., development idiosyncratic deals and flexible idiosyncratic deals; Rousseau et al., 2006), including special training opportunities, flexible work schedules, telecommuting opportunities, and tailored compensation packages. Previous research has shown that social exchange theory can effectively explain the myth of idiosyncratic deals (Hornung et al., 2010; Rosen et al., 2013). In this study, distributed leadership meets the social exchange norms of bilateral leadership (leader and employees), interaction (mobility to assume leadership responsibilities in a dynamic context), and reciprocity (meeting the needs of leaders and employees). Therefore, we expect to explain that distributed leadership can promote mutually beneficial idiosyncratic deals between leaders and employees from the perspective of social exchange theory, and further induce new generation employees’ proactive behavior. Distributed leadership implies “actively brokering, facilitating, and supporting the leadership of others” (Harris, 2013). This research believes that distributed leadership is conducive to the achievement of idiosyncratic deals. First, in a distributed context, the leader provides resources (leadership) to employees, and employees give back resources (professional knowledge and skills) to the leader. According to social exchange theory, this exchange relationship will evolve into trust, loyalty, and mutual commitment over time (Cropanzano and Mitchell, 2005), and shape an open and trusting organizational culture (Heikka et al., 2013), which provides a basis for the relationship and culture for leaders and employees to equally negotiate idiosyncratic deals. Second, Johnson (2004) pointed out that distributed leadership can be regarded as an organizational resource, but a pure organizational perspective can easily fall into “new managerialism” and reduce organizational efficiency. It is also necessary to realize the organization’s daily operation from the perspective of individuals (agents). Therefore, the interaction between leaders and followers in the context of the situation is crucial to the practice of distributed leadership. When leaders are supportive and considerate, employees are more likely to successfully negotiate idiosyncratic deals regarding career development opportunities and scheduling flexibility (Hornung et al., 2011). Third, Woods (2004) pointed out that distributed leadership is democratic, and democratic leadership can use individual potential to serve the organization. The new generation of employees in a democratic atmosphere is more likely to believe that their ideas and suggestions will be more easily accepted and adopted by the organization or the leader (Frese et al., 1997). In other words, distributed leadership provides space for the implementation of idiosyncratic deals. Finally, in “distributed” practice, organizations and managers need to strive to achieve higher efficiency, flexibility, and competitiveness (Thorpe et al., 2011). Faced with the above pressure, Chernyak-Hai and Rabenu (2018) proposed that organizations should adapt to the diversified labor force of the “new era” and introduce a unique human resource management approach that can consider different employee preferences and abilities. Previous organizations have implemented idiosyncratic deals to adapt to the individual situation of their employees (Rousseau et al., 2006). Thus, we propose the following hypothesis:

*Hypothesis* 2: Distributed leadership has a positive impact on idiosyncratic deals.

### Mediating Role of Idiosyncratic Deals

Idiosyncratic deals not only meet the personalized psychological needs of new generation employees (Hornung et al., 2008), but also improve their precepted organization image and evaluation (Ng and Feldman, 2010), and ultimately encourage them to undertake proactive behavior (Huo et al., 2014). For example, in terms of psychological needs, idiosyncratic deals enhance employees’ competence, autonomy, and relationship satisfaction, and promote employees’ proactive professional behaviors. For the perception of organizational image and evaluation, idiosyncratic deals can promote employees’ proactive behavior through perceived organizational support and organizational-based self-esteem (Liu et al., 2013). Considering the autonomous work values of new generation employees, this study believes that the contextual characteristics of distributed leadership (bilateral, interactive, and reciprocal) are conducive to the negotiation of idiosyncratic deals between leaders and new generation employees, which in turn stimulates new generation employees’ proactive behavior.

First, the organization signs idiosyncratic deals with a new generation employee, indicating that the organization respects the subjective preference of the employee for work content, and accepts that the employee makes more efforts in the direction of their own identity (Strauss et al., 2012). In other words, this kind of work autonomy is conducive to promoting new generation employees’ proactive behavior for improving themselves. Second, for an organization, idiosyncratic deals are low-cost incentives that can indirectly motivate employees who advocate the organization’s vision (Owens and Hekman, 2012), and inspire employees to engage in positive behaviors to improve the organizational environment. Finally, idiosyncratic deals allow employees to reorganize and configure various work tasks and resources, providing organizational support for employees’ proactive behavior. For example, Hornung et al. (2010) mentioned that idiosyncratic deals could improve work flexibility and controllability, reduce work pressure, and promote employees’ proactive tendency and work participation.

In summary, social exchange theory suggests that employees who obtain value and benefits from the organization will engage in proactive behavior conducive to achieving organizational goals. Thus, this study believes that distributed leadership has an impact on new generation employees’ proactive behavior through idiosyncratic deals, proposing the following hypothesis:

*Hypothesis* 3: Idiosyncratic deals will mediate the effect of distributed leadership on new generation employees’ proactive behavior.

### Distributed Leadership and Meaningfulness of Work

Previous studies have widely argued that the meaningfulness of work includes “bipartite value” (subjectivity and objectivity; Yeoman, 2014), and three dimensions (positive meaning at work, meaning creation at work, and strong friendly motivation; Steger et al., 2013). In this study, we formally define “meaningfulness of work” as employees’ beliefs that their work has at least one distinct purpose that they also consider personally significant.

Self-enhancement depicts that people generally have a tendency to maximize their good experience and make positive evaluation of their experiences (Alicke and Sedikides, 2009), which not only enhances individuals’ psychological benefits (Kurman, 2003), but also affects individuals’ self-conception (Hideg and Ferris, 2014), attitudes, and emotions (Dufner et al., 2019). According to self-enhancement theory (Korman, 2012), an employee’s self-enhancement motivation is most likely when he or she is in a work environment that provides both the opportunity and the encouragement to attain goals that reflect positive self-feelings and individual effort. For new generation employees, distributed leadership is conducive to evaluating the importance and value of their work, enhancing their sense of meaningfulness of work.

First, new generation employees have a psychological need for self-vale (Yang and Yang, 2020). Distributed leadership behavior indirectly shows that the organization is confident that the employee can apply his or her new skills/knowledge for the betterment of the organization. This kind of affirmation and respect from the organization will make new generation employees believe that they are individuals with independent meanings, increasing their meaningfulness of work. Second, affected by the development of the internet and the Chinese one-child policy, new generation employees often have a relatively vague job role position after entering the workplace (Zhu and Warner, 2018). Distributed leadership helps employees establish a clear career development direction. As new generation employees’ sense of work direction and purpose continues to be clear, their subjective feelings and psychological experience of work will be more positive, which indirectly promotes the improvement of meaningfulness of work. Finally, new generation employees yearn for a positive working relationship and gain the trust and understanding of those around them (Xu et al., 2020). In this vein, distributed leadership can establish an atmosphere of trust between leaders and employees, encouraging new generation employees to actively cooperate, communicate, and share with each other. This positive working relationship is more likely to increase new generation employees’ meaningfulness of work (Bailey et al., 2019). Thus, we propose the following hypothesis:

*Hypothesis* 4: Distributed leadership has a positive impact on meaningfulness of work.

### Mediating Role of Meaningfulness of Work

Self-enhancement theory suggests that when employees perceive themselves as important and of valuable positive signals from the organization, they may become more engaged and motivated to actively solve organizational problems (Liu et al., 2013). As a psychological experience of new generation employees, meaningfulness of work has a positive predictive effect on their proactive behavior. First, individuals can better obtain resources from the environment to achieve their goals and values when environmental attributes can better meet their psychological needs (Kristof-Brown et al., 2005). Employees who perceive positive psychological meanings from their work will respond positively to the environment (Lam et al., 2016). Second, the meaningfulness of work and life is always inseparable, and work is an important source of meaning in life (Steger et al., 2013). To be able to incorporate the meaningfulness of work into our lives, we must become valuers, that is, cocreators of values and meanings. Previous studies have shown that work can help individuals deepen their understanding of themselves and the world around them, promote their growth, and provide psychological capital for changing the environment and themselves (Allan et al., 2016). Finally, new generation employees have a strong and friendly motivation. They always try to achieve their greater value and make broader contributions to undertake positive behavior, such as introducing new working methods, improving work processes, actively seeking feedback, and influencing organizational strategies (Furstenberg et al., 2020).

In summary, distributed leadership promotes new generation employees’ proactive behavior by increasing their meaningfulness of work. On the one hand, distributed leadership is open and equal to each team member’s suggestions and ideas, which prompts employees to believe that they are individuals with independent meaning, thereby generating higher intrinsic motivation and actively engaging in work (Tu et al., 2020). On the other hand, distributed leadership respects the value and contribution of each team member. Employees will have a strong sense of identity and belonging when they are respected in the work environment. In turn, new generation employees will show repay for the organization in positive behavior. Therefore, we propose the following hypothesis:

*Hypothesis* 5: Meaningfulness of work will mediate the effect of distributed leadership on new generation employees’ proactive behavior.

### A Multistep Mediation Model

Self-enhancement is a common need in individuals (Dufner et al., 2019), which reflects that individual will be more positive in evaluating their value in the desired characteristic dimension (Cai et al., 2011). Organizational context and work forms are important exogenous factors affecting individuals’ self-enhancement (Ajzen, 2002; Korman, 2012). In this research, we believe that idiosyncratic deals, as a representation of a new work form, positively impact the psychological perception of new generation employees’ work significance.

Idiosyncratic deals can bring positive work results to organizations (Singh and Vidyarthi, 2018), enhancing the meaningfulness of work for new generation employees. First, flexibility idiosyncratic deals refer to special arrangements for work schedules (Hornung et al., 2010), which provides a space for coordination of work-family related issues and reduces work-family conflicts (Hornung et al., 2014). In other words, flexible idiosyncratic deals can meet the needs of new generation employees for work-life balance, and enhance their positive experience of meaning in life. Second, as for the formation mechanism of meaningfulness of work, job design is an important influencing factor of meaningfulness of work, and optimized work design methods can create more rewards and meaning (Hornung et al., 2019). Idiosyncratic deals, as a supplement and adjustment to traditional “top-down” and “bottom-up” work design methods (Grant and Parker, 2009; Hornung et al., 2010), are bound to affect individuals’ perception of meaningfulness of work. Finally, self-enhancement theory shows that individuals with self-enhancement motives will always attract group members that are friendly to them. In the workplace, being kind to others often leads to positive working relationships, which may influence members’ meaningfulness of work (Bailey et al., 2019). Therefore, idiosyncratic deals have an impact on meaningfulness of work for new generation employees.

Based on the above discussion, we believe that there is a multistep process “distributed leadership→ idiosyncratic deals-meaningfulness of work→ proactive behavior.” Therefore, we propose the following hypothesis:

*Hypothesis* 6: Idiosyncratic deals will enhance new generation employees’ proactive behavior both directly and indirectly through increased meaningfulness of work.

## Materials and Methods

### Sample and Procedure

The samples in this study were from the same one company. And the differences between units within the company were small. Therefore, we obtained data through cluster sampling technique. To minimize the impact of common method variance (CMV) on the research conclusions (Podsakoff et al., 2003), this research designed a supervisor-employee paired questionnaire, using a three-point survey method to ensure that the research reached a scientific and reliable conclusion. At the same time, based on previous studies (Fida et al., 2018), there is a 1-month interval between each data collection. Before the formal survey, the research team negotiated with the research company to explain the research details. This survey was anonymous, the research results were only used for scientific research, and we promised to keep the information obtained in the research strictly confidential. Finally, we two parties communicated the time for data collection. First, we marked the code for supervisors and new generation employees; then, they filled out the paper questionnaire, and only those who completed the previous survey could enter the next survey. Finally, the completed questionnaire was placed in a sealed envelope and collected by the staff. In the end, the sample of this study included 304 new-generation employees and 26 supervisors, and the effective recovery rates of the two were 87.61 and 83.33%, respectively. At time 1, new generation employees filled out the basic information and distributed leadership questionnaire; at time 2, new generation employees filled in the personalized work agreement and work meaning questionnaire; and at time 3, supervisors evaluated the proactive behavior of new generation employees. Among the 304 new generation employees, 65.37% were women, 58.05% were aged 18–25 years, 55.12% had a college degree, and 68.29% had worked less than 3years.

### Measures

To improve the accuracy of expression, based on existing research (Brislin, 1970), each measurement item of key variables has been carefully modified in accordance with the translation and back-translation procedures. In this survey, new generation employees assessed three variables (i.e., distributed leadership, idiosyncratic deals, and meaningfulness of work), and supervisors assessed new generation employees’ proactive behavior. A five-point Likert scale was used for the four variables scales, ranging from 1 (strongly disagree) to 5 (strongly agree).

Distributed leadership was measured through a 20-item scale (Hairon and Goh, 2015). A sample items is “My leader cares about the opinions of subordinates.” Cronbach’s α was 0.876. A six-item scale was adapted to measure idiosyncratic deals (Hornung et al., 2008). A sample items is “The start and the end time of work can be adjusted flexibly.” Cronbach’s α was 0.857. The meaningfulness of work was measured with a five-item scale (Bunderson and Thompson, 2009). A sample items is “The work I do is important.” Cronbach’s α was 0.890. The supervisors were required to evaluate the new generation employees’ proactive behavior through seven-item scale (Frese et al., 1997). A sample item is “I actively solve problems.” Cronbach’s α was 0.929.

### Control Variables

Following previous studies (e.g., Fang et al., 2019), we controlled for demographic variables, such as participants’ gender, age, education, and job tenure that may influence proactive behavior.

## Results

### Data Analysis

According to the research of Naz et al. (2021), PLS-SEM can be used as an effective technique for evaluating models, while avoiding the problems of data normality and sample size. Therefore, we used Smart PLS 3 to test the hypothesis. In addition, we also used PLS algorithm and bootstrapping method to test internal consistency reliability, path coefficients, and mediation effects.

### Common Method Variance Test

We used the CMV test to avoid possible bias. First, we adopted the method of supervisor-employee pairing in the research design, and tracked the questionnaire data longitudinally at three-time points. Second, we conducted a confirmatory factor analysis by applying the Harman single-factor test. The result shows that the variance contribution rate explained by the first principal component was 15.859%, which did not exceed the standard 40%. Third, according to research design of Podsakoff et al. (2003), we further controlled for an unmeasured latent “method” factor to confirm Harman’s single-factor test. Finally, according to the research of Kock (2015), existing work attempts to estimate CMV by the variance inflation factor (VIF) calculated by the complete collinearity test. When the VIF score is higher than 3.3, it means that the estimation model may be accompanied by CMV problems. The results of this study showed that the VIF scores of the four potential variables were all lower than 3.3, thus claimed that the data were not contaminated by the errors of CMV. As shown in Table 1, the four-factor model provided a better model fit than any other competition model, indicating that CMV was within an acceptable range.

**Table 1:** Analysis of confirmatory factors of competition model.

Model	*χ*^2^/df	GFI	RMSEA	RMR	CFI	TLI	IFI	SRMR
Four factors (DL. ID. MW. PB)	1.683	0.846	0.058	0.044	0.937	0.928	0.938	0.048
Three factors (DL + ID. MW. PB)	2.872	0.694	0.096	0.066	0.796	0.780	0.798	0.068
Two factors (DL + ID + MW. PB)	3.405	0.649	0.109	0.075	0.737	0.717	0.739	0.077
Single factor (DL + ID + MW + PB)	3.721	0.623	0.115	0.079	0.701	0.680	0.704	0.081

*N* = 304. “+” represents two factors merged into one. DL, distributed leadership; ID, idiosyncratic deals; MW, meaningfulness of work; and PB, proactive behavior.

### Confirmatory Factor Analysis

Table 1 shows the results of confirmatory factor analysis. Among them, the four-factor model has the best model fit: *χ^2^/df* = 1.683; GFI = 0.846; RMSEA = 0.058; RMR = 0.044; CFI = 0.937; NNFI = 0.928; TLI = 0.928; IFI = 0.938; and SRMR = 0.048. Therefore, the four-factor model has a good discriminative validity, which provides favorable support for further hypothesis testing.

### Descriptive Statistics and Correlations

Table 2 presents all the variables’ descriptive statistics and correlations. According to the previous research (Fornell and Larcker, 1981; Naz et al., 2020), all the square roots of AVE for the constructs are bigger than the off-diagonal elements or coefficients in the relative columns, hence, confirming an indication of discriminant validity. The results in Table 2 proved the satisfactory discriminant validity. As predicted distributed leadership is significantly correlated with idiosyncratic deals (*r* = 0.658, *p* < 0.01), meaningfulness of work (*r* = 0.615, *p* < 0.01), and proactive behavior and proactive behavior (*r* = 0.710, *p* < 0.01). Concerning control variables, distributed leadership was correlated with education. Otherwise, idiosyncratic deals and meaningfulness of work were correlated with job tenure.

**Table 2:** Statistical table of mean, SD, and correlation coefficient.

Variable	1	2	3	4	5	6	7	8
Gender								
Age	−0.043							
Education	0.117^*^	−0.009						
Job tenure	−0.079	0.604^**^	−0.305^**^					
DL	−0.032	−0.015	0.009	−0.041	**0.732**			
DI	−0.083	−0.023	0.025	−0.030	0.658^**^	**0.715**		
MW	−0.075	0.131^*^	−0.061	0.073	0.615^**^	0.589^**^	**0.812**	
PB	−0.059	0.086	0.061	−0.002	0.710^**^	0.612^**^	0.681^**^	**0.739**
M	1.652	2.529	3.333	2.147	3.517	3.334	3.493	3.627
SD	0.478	0.827	0.678	1.382	0.721	0.737	0.853	0.687

*N* = 304. The diagonally bolded number is the square root value of AVE. DL, distributed leadership; ID, idiosyncratic deals; MW, meaningfulness of work; and PB, proactive behavior.

* *p*<0.05;

** *p*<0.01 (two-sided detection).

### Hypotheses Testing

To test our hypotheses, we used a hierarchical regression model in MPLUS 7.8. As shown in Table 3, distributed leadership positively correlated with new generation employees’ proactive behavior (Model 6: *β* = 0.674, *p* < 0.01) after controlling for participants’ gender, age, education, and job tenure, thus supporting hypothesis 1.

**Table 3:** Results of hierarchical regression model.

	DI			MW			PB		
	Model 1	Model 2	Model 3	Model 4	Model 5	Model 6	Model 7	Model 8	Model 9
Constant	3.508^**^	1.066^**^	3.615^**^	0.961^*^	3.424^**^	0.968^**^	0.715^*^	0.666^*^	0.560^*^
Gender	−0.136	−0.100	−0.115	−0.075	−0.093	−0.057	−0.033	−0.033	−0.023
Age	−0.014	−0.024	0.161	0.150	0.103	0.093	0.098	0.046	0.054
Education	0.032	0.034	−0.083	−0.081	0.050	0.053	0.045	0.078	0.071
Tenure	−0.010	0.010	−0.029	−0.008	−0.033	−0.014	−0.016	−0.011	−0.013
DL		0.671^**^		0.729^**^		0.674^**^	0.515^**^	0.445^**^	0.383^**^
DI							0.237^**^		0.131^*^
MW								0.314^**^	0.279^**^
R^2^	0.009	0.438	0.026	0.404	0.018	0.518	0.554	0.609	0.619
Adjust R^2^	−0.011	0.424	0.006	0.389	−0.002	0.506	0.541	0.597	0.605
F value	0.446	30.868^**^	1.316	26.846^**^	0.892	42.516^**^	40.814^**^	51.045^**^	45.400^**^

N = 304. DL, distributed leadership; ID, idiosyncratic deals; MW, meaningfulness of work; and PB, proactive behavior.

* *p*<0.05;

** *p*<0.01 (two-sided detection).

As displayed in Table 3, we determined that distributed leadership had a positive direct relationship with idiosyncratic deals (Model 2: *β* = 0.671, *p* < 0.01) and meaningfulness of work (Model 4: *β* = −0.729, *p* < 0.01), after controlling for employees’ gender, age, and job tenure, thus supporting hypothesis 2 and hypothesis 4.

Hypothesis 3 and hypothesis 5 suggested that idiosyncratic deals and meaningfulness of work will mediate the influence of distributed leadership on new generation employees’ proactive behavior. As shown in Table 3, the positive effect of distributed leadership on proactive behavior has been weakened, but it is still significant after adding idiosyncratic deals (Model 7: *β* = 0.237, *p* < 0.01). Similarly, the positive effect of distributed leadership on proactive behavior has been weakened, but it is still significant after adding meaningfulness of work (Model 8: *β* = 0.314, *p* < 0.01). hypothesis 3 and hypothesis 5 received support.

Following the Hayes PROCESS macro (Hayes, 2017), we applied bootstrapping (bootstrap sample size = 10,000) to obtain the bias-corrected CI to establish the significance of the mediation. Bootstrap results are shown in Table 4. The indirect effect of idiosyncratic deals is significant (*B* = 0.088, CI = [0.117, 0.162]), and the indirect effect of meaningfulness of work is significant (*B* = 0.132, CI = [0.080, 0.275]). Finally, the effect of distributed leadership on new generation employees’ proactive behavior mediated by idiosyncratic deals and meaningfulness of work is significant (*B* = 0.071, CI = [0.047, 0.177]), hypothesis 6 received support.

**Table 4:** Analysis results of multi-chain mediation.

Path	Direct effect			Indirect effect		
	Effect size	95% CI		Effect size	95% CI	
		LL	UP		LL	UP
Path 1: DL→DI→PB	0.383^**^	0.265	0.502	0.088^**^	0.117	0.162
Path 2: DL→MW→PB				0.132^**^	0.080	0.275
Path 3: DL→DI→MW→PB				0.071^**^	0.047	0.177

DL, distributed leadership; ID, idiosyncratic deals; MW, meaningfulness of work; PB, proactive behavior; LL, lower level; UL, upper level; and CL, confidence interval.

* *p*<0.05;

** *p*<0.01 (two-sided detection).

### Competitive Models Analysis

This study used a competitive model strategy to verify further verify the adaptability of the research data and the theoretical model, and six competitive models were obtained, of which M1 is the initial theoretical model of this research. As shown in Table 5, the initial model M1 has the highest model fit. First, the load values of each factor in M1 were all >0.6 and <0.9, reaching a significant level (*p* < 0.01). Second, *x^2^/df* = 1.943 < 3, RMSEA = 0.079 < 0.1, and RMR = 0.021 < 0.05, indicating that M1 fits well. Third, GFI = 0.977, CFI = 0.966, NFI = 0.965, and IFI = 0.971, all >0.9; review index, AIC = 60.000, and BIC = 60.120, which is smaller than the corresponding value of any other competition model. Overall, all the hypotheses of this study have been supported.

**Table 5:** Comparative analysis of competitive models.

Model	*χ^2^*/df	RMSEA	RMR	GFI	CFI	NFI	IFI	AIC	BIC
M1	1.943	0.079	0.021	0.977	0.966	0.965	0.971	60.000	60.120
M2	6.921	0.435	0.068	0.920	0.878	0.878	0.879	96.921	97.030
M3	4.765	0.357	0.430	0.943	0.918	0.917	0.918	85.765	85.874
M4	5.425	0.638	0.148	0.856	0.738	0.738	0.739	99.425	99.534
M5	5.398	0.248	0.026	0.971	0.960	0.960	0.961	70.398	70.506
M6	3.178	0.273	0.026	0.965	0.952	0.952	0.952	81.178	81.287
M7	5.883	0.244	0.039	0.971	0.962	0.961	0.962	68.883	68.991

**Figure 1:**
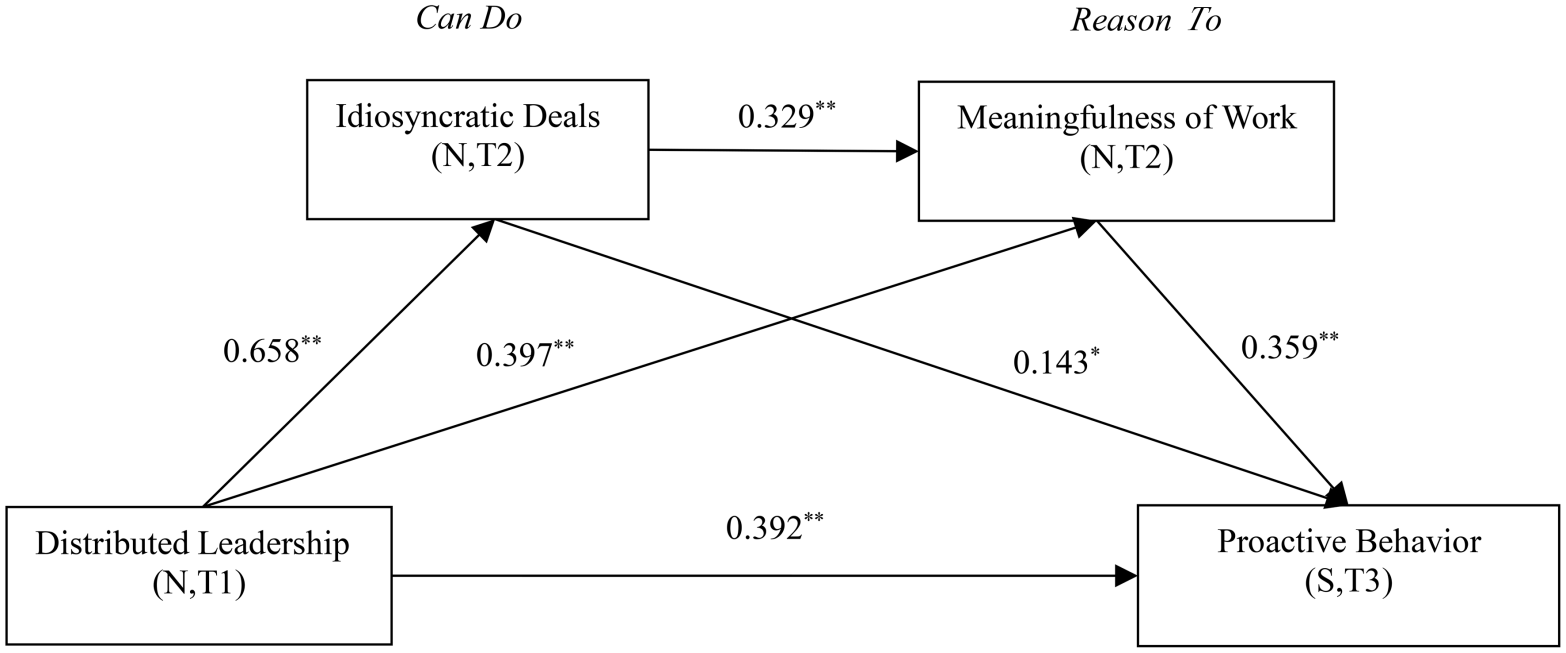
Diagram of the path coefficients of the multistep mediation model. N = New generation employees; S = Supervisor. T1 = Time 1; T2 = Time 2; and T3 = Time 3. ^*^p < 0.05; ^**^p < 0.01 (two-sided detection).

PLS-SEM can better handle measurement errors and provide a more accurate evaluation of intermediary relationships. In addition, PLS path analysis enables itself to fully meet the needs of application solutions, and it is relatively more advantageous to use in complex research (Naz et al., 2020). We used the software Smart PLS 3 to draw Figure 1. As shown in Figure 1, we drew a path diagram of the multistep mediation model.

## Discussion

We developed a multistep mediation model to examine how distributed leadership influences new generation employees’ proactive behavior through the integrated perspective of social exchange theory and self-enhancement theory. The empirical results showed that distributed leadership was positively related to new generation employees’ proactive behavior, which was mediated by idiosyncratic deals, meaningfulness of work, and their multistep mediation. First, distributed leadership had a positive impact on employee behavior, which was consistent with the research of Canterino et al. (2020). Moreover, we focused our research samples on new generation employees. Fang et al. (2019) explored the impact of inclusive leadership on new generation employees’ innovative behavior, but they did not pay enough attention to distributed leadership. To a certain extent, we provided a supplement to the previous conclusions. Second, previous studies focused on the impact of idiosyncratic deals on employees’ attitudes and behaviors (Liao et al., 2017; Singh and Vidyarthi, 2018), while ignoring the effect of idiosyncratic deals in the relationship between leadership styles and new generation employees’ behavior. We provided fresh inspiration for research literature related to idiosyncratic deals. Third, meaningfulness of work was one of the psychological paths that the distributed leadership style effected new generation employees’ proactive behavior. This conclusion was basically consistent with Liu et al. (2013). Recently, some scholars have paid attention to the modular meaning and mediating role of meaningfulness of work (Landells and Albrecht, 2019; Schnell and Hoffmann, 2020). However, they did not discuss the role of meaningfulness of work between leadership style and employee proactive behavior. In fact, new generation employees could effectively recognize their unique value through voluntary, equal and reciprocal work agreements, and then enhance the meaningfulness in their work. Finally, idiosyncratic deals and meaningfulness of work played a multistep mediation role between distributed leadership and new generation employees’ proactive behavior. This conclusion extends the research of Hornung et al. (2011). And it supports the view that signing idiosyncratic deals between leaders and employees would not only help organizational members improve their own behavior, but also help employees build positive self-concepts as independent individuals.

### Theoretical Implications

This study provides several theoretical contributions. First, our findings point to the positive effect of distributed leadership in predicting new generation employees’ proactive behavior. Previous studies regarding distributed leadership mainly focus on education management (Liu et al., 2021; Or and Berkovich, 2021). Gradually, it is being favored by scholars in the field of organization management (Lumby, 2013), with a positive impact on individuals’ proactive behavior (Butler and Tregaskis, 2018; Amels et al., 2020; Canterino et al., 2020), team behavior (Mehra et al., 2006; Wang et al., 2014), and organization performance (Liu and Werblow, 2019). Furthermore, previous studies have mostly explored the influence of servant leadership (Song et al., 2021) and authentic leadership (Hu et al., 2018) on employees’ proactive behavior. The empirical research on the influence of distributed leadership on employees’ behavior is very insufficient. It is worth noting that Fang et al. (2020) discussed the influence of leadership style on the behavior of the new generation of employees, he focused on the negative behavior (resignation behavior) of new generation employees, not positive behavior (proactive behavior). Our research has found the antecedents of the new generation of employees’ proactive behavior (distributed leadership) from the leadership style, and provided a new perspective for inspiring the new generation of employees’ proactive behavior.

Second, we explored how distributed leadership influenced the proactive behavior of new generation employees from the perspective of social exchange, which helped us better understand the mediation of idiosyncratic deals. Previous studies have confirmed the impact of idiosyncratic deals on employees’ attitudes and behaviors from the perspective of social exchange (Liao et al., 2017; Singh and Vidyarthi, 2018; Wang et al., 2019). In terms of behavior, scholars are mainly concerned with voice behavior (Ng and Feldman, 2015), organizational citizenship behavior (Huo et al., 2014), and creativity (Wang et al., 2018). Empirical analysis of the proactive behavior of new generation employees is not sufficient. In addition, a few scholars have examined the mediation of job characteristics, social exchange, and self-improvement between idiosyncratic deals and proactive behavior (Hornung et al., 2010; Liu et al., 2013). However, they have ignored the transmission effect of idiosyncratic deals between distributed leadership and new generation employees’ proactive behaviors. This research enriched and expanded the literature on the relationship between distributed leadership and new generation employees’ proactive behavior by adding a substantial intermediary variable.

Third, this research responded to the viewpoint of Hornung et al. (2019) which based on the “micro-emancipatory” actions employees engage in, “bottom-up” processes create more rewarding and meaningful work experiences. It further confirmed the conclusion that idiosyncratic deals were always related to a positive work experience (e.g., meaningfulness of work). Previous research mainly used intermediary mechanisms, such as organizational-based self-esteem (Wu et al., 2019), psychological capital (Hu et al., 2018), and autonomous psychological needs (Chen et al., 2021) to study the intermediary mechanism of leadership or organizational context on employee behavior. However, from the perspective of self-enhancement, this research provided a new idea for explaining the proactive behavior of new generation employees. Distributed leadership stimulates the intrinsic motivation of new generation employees by exchanging leadership with new generation employees, enhances their self-worth and sense of work meaning, and then promotes their proactive behavior. This discovery not only opened the black box of the new generation of employees’ proactive behavior from the perspective of employee psychological experience, but also cleverly responded to emphasis of Parker et al. (2010) on the “reason to” and “can do” of the proactive behavior motivation model.

### Practical Implications

Our study advances the idea that it is important to practice distributed leadership to enhance new generation employees’ idiosyncratic deals, meaningfulness of work, and proactive behavior. First, our research clearly illustrates the influence of distributed leadership on the proactive behavior of new generation employees. Therefore, organizations should focus on cultivating distributed leadership and exerting the effectiveness of this leadership style in management practice. For organizational managers, the direction of leadership distribution should be carefully analyzed. Especially in the period of organizational change, managers can use distributed leadership as a strategic choice, which can help overcome the shortcomings of the pyramidal leadership structure, and better tap and release the management potential of their subordinates. At the same time, managers should also create an organizational atmosphere of openness, trust, and cooperation, and explore democratic and equal participation and decision-making mechanisms, such as brainstorming and discussion methods. Therefore, organizations should cultivate and value the positive effects of distributed leadership internally.

Second, idiosyncratic deals directly or indirectly affected the relationship between distributed leadership and new generation employees’ proactive behavior. In other words, organizations should reasonably use the advantages of idiosyncratic deals in attracting, retaining, and motivating talent. For new generation employees, they had a psychological need for autonomy and respect. Managers could use idiosyncratic deals to promote and improve the resolution of complex problems, such as arranging personalized career development training for new generation employees, or regularly renegotiating work benefits with new generation employees. In addition, new generation employees pay attention to the cultivation of personal interests and work-life balance, and managers could allow new generation of employees to work remotely through negotiation. In particular, it was necessary to give play to the role of distributed management in promoting idiosyncratic deals, thereby enhancing the proactive behavior of new generation employees.

Third, distributed leadership influenced the proactive behavior of new generation employees through the meaningfulness of work. Organizations should pay attention to this discovery, encourage leaders and employees to exchange roles, assume part of the power and responsibilities within the team, and scientifically and reasonably play the role of distributed leadership in enhancing the significance of new generation employees. New generation employees have the basic needs of pursuing the meaning of work. They can enhance their sense of work meaning by gaining freedom, autonomy, and dignity in the organization. Therefore, organizations should provide employees with organizational culture, working environment, and benefits related to decent work. At the same time, the organization should adjust and improve the internal work design, give employees the necessary work autonomy, and appropriately enhance the challenges brought about by work tasks, which can effectively enhance the sense of accomplishment of employees, and cultivate and intervene in the meaningfulness of work for new generation employees.

### Limitations and Future Research Directions

Although our research has certain theoretical contributions and practical implications, there are some associated limitations that might be addressed by future research.

First, this research relied only on one Chinese new energy vehicle company, which may reduce the external validity of research conclusions. Although data support all research hypotheses, this research model may not be applicable to other countries’ organizations. In the future, we should add research samples from Western countries with cultural differences and perform a cross-cultural study to make the research conclusions universal worldwide.

Second, this research only explored the multistep mechanism and no moderating roles were considered, which obviously ignored the influence of contextual factors. Employees’ responses to distributed leadership may vary significantly when they work in different social culture and organizational culture. Future research needs to examine the impact of social and cultural boundary conditions on distributed leadership and new generation employees’ proactive behavior.

Third, there may be many specific forms of proactive behaviors in the workplace. This research did not focus on one specific proactive behavior, such as proactive change behaviors, proactive innovation behaviors, and helping behaviors. Future researches might specifically examine how distributed leadership affects other certain forms of new generation employees’ proactive contribution, which could effectively deepen the conclusions of this research.

## Conclusion

The motivation for new generation employees’ proactive behavior largely depends on the leadership style of the leader. This study provides new insight into the relationship between distributed leadership and the proactive behavior of new generation employees and helps us better understand the impact of distributed leadership on proactive behavior. First, based on the integrated perspective of social exchange and self-enhancement, we constructed a multistep mediation theoretical model to explore the influence mechanism of distributed leadership on the proactive behavior of new generation employees. In China’s organizational context, we find that distributed leadership has a positive effect on proactive behavior of new generation employees. Second, idiosyncratic deals play a positive role in the relationship between distributed leadership and proactive behavior of new generation employees. This finding further deepens our understanding of the “can do” path between distributed leadership and proactive behavior of new generation employees. Third, in addition to “can do” path, meaningfulness of work lies in integrating the cognitive and evaluation aspects and plays a positive role in the relationship between distributed leadership and proactive behavior of new generation employees. This finding further deepens our understanding of the “reason to” path between distributed leadership and proactive behavior of new generation employees.

## Data Availability Statement

The raw data supporting the conclusions of this article will be made available by the authors, without undue reservation.

## Ethics Statement

The studies involving human participants were reviewed and approved by The studies involving human participants were reviewed and approved by the Ethics Committee of Shanghai University. All participants provided active informed consent. Written informed consent for participation was not required for this study in accordance with the national legislation and the institutional requirements.

## Author Contributions

SX and HZ designed the study and wrote the paper. YD collected the data and wrote the paper. LL analyzed the data. SX and JM revised and edited the manuscript. All authors contributed to the article and approved the submitted version.

## Funding

This study was supported by National Natural Science Foundation of China (grant no. 71872111) and Humanities and Social Science Research Planning Fund Project of the Ministry of Education (grant no. 16YJA630036).

## Conflict of Interest

The authors declare that the research was conducted in the absence of any commercial or financial relationships that could be construed as a potential conflict of interest.

## Publisher’s Note

All claims expressed in this article are solely those of the authors and do not necessarily represent those of their affiliated organizations, or those of the publisher, the editors and the reviewers. Any product that may be evaluated in this article, or claim that may be made by its manufacturer, is not guaranteed or endorsed by the publisher.
